# Oral Combined Probiotics *Clostridium butyricum* and *Akkermansia muciniphila* Inhibits the Progression of 4T1 Breast Cancer by Activating Bcl‐2/Bax Pathway

**DOI:** 10.1002/cam4.70987

**Published:** 2025-06-11

**Authors:** Xiaowei Li, Dengxiong Hua, Daoyan Wu, Wei Hong, Yingqian Kang, Lei Tang, Qin Yang, Xinxin Wang, Boyan Li, Renmin Li, Zhenghong Chen, Guzhen Cui

**Affiliations:** ^1^ Key Laboratory of Microbiology and Parasitology of Education Department of Guizhou & Key Laboratory of Medical Molecular Biology of Guizhou Province Guizhou Medical University Guiyang China; ^2^ Joint Laboratory of Helicobacter Pylori and Intestinal Microecology of Affiliated Hospital of Guizhou Medical University Guiyang China; ^3^ Guizhou Key Laboratory of Microbio and Infectious Disease Prevention & Control Guiyang China; ^4^ Guizhou Provincial Engineering Technology Research Center for Chemical Drug R&D Guizhou Medical University Guiyang China; ^5^ School of Public Health Guizhou Medical University Guiyang China; ^6^ Standard Testing Group Co., Ltd. Qingdao China

**Keywords:** *Akkermansia muciniphila*, apoptosis, Bcl‐2/Bax signaling pathway, breast cancer, *Clostridium butyricum*, probiotics

## Abstract

**Background:**

Breast cancer is the most common malignant tumor among women. Recent studies have found that gut probiotics and their metabolic products play a significant role in activating the immune system, reshaping the tumor microenvironment, and inhibiting cancer progression.

**Methods:**

We established a 4T1 tumor‐bearing mice model and analyzed the proportions of CD4^+^ T and CD8^+^ T cells in the spleen using flow cytometry and immunohistochemistry. The expression levels of TNF‐α, IL‐6, and IL‐10 were measured by enzyme‐linked immunosorbent assay. Hematoxylin–eosin staining was used to observe the tumor morphology. Selective protein blotting and quantitative real‐time PCR were used to analyze the expression of Bax, Bcl‐2, and Caspase‐3. Cell proliferation was evaluated using the MTT assay, and apoptosis was detected by flow cytometry.

**Results:**

The results indicated that oral administration of CB and AKK possesses the capability to inhibit the progression of 4T1 breast cancer; however, the combined treatment with both strains (CB‐AKK) exhibited significantly superior effects compared to each individual strain. Further mechanistic analysis revealed that the CB‐AKK combination could activate the antitumor immunity in mice and reshape the tumor microenvironment. Additionally, it was found that the live bacteria and their metabolites derived from CB‐AKK could inhibit cell proliferation and promote tumor apoptosis by activating the Bcl‐2/Bax signaling pathway.

**Conclusion:**

This study is the first to demonstrate that orally administered live bacteria CB‐AKK can inhibit the progression of 4T1 breast cancer, providing a promising new strategy for the development of innovative biotherapies for breast cancer.

## 1 Introduction

Breast cancer is the most common and highest‐incidence malignant tumor among women [1]. According to global cancer data for 2022, there are approximately 2.3 million new cases of breast cancer, making it the second most prevalent type of cancer after lung cancer, with its incidence increasing annually worldwide [2]. In addition to conventional treatments, such as surgical resection and radiotherapy/chemotherapy, biological therapies have garnered significant popularity and attention in recent years due to their advantages of minimal side effects, enhanced patient experience, and high acceptance, gradually becoming one of the emerging options for cancer treatment [3, 4].

Intestinal probiotics and their metabolites reshape the tumor microenvironment by activating the body's immune system (both innate and adaptive immunity), demonstrating significant efficacy in preventing tumor development and inhibiting tumor progression [5, 6, 7]. Researchers have currently developed various types of oncolytic probiotics with anticancer properties [8]. For example, engineered probiotic *Escherichia coli* Nissle (EcN) and its outer membrane vesicles have been developed as effective antitumor vaccines, exhibiting significant efficacy in the treatment of breast cancer [9], colon cancer [10, 11], and melanoma [12]. *Roseburia intestinalis* enhances anti‐PD‐1 efficacy in colorectal cancer by activating cytotoxic CD8^+^ T cells [13]. *Lactobacillus johnsonii* and *Clostridium sporogenes* collaborate to produce indole‐3‐propionic acid, enhancing the antitumor effect of immune checkpoint blockade in pan‐cancer [14]. In addition, certain intracellular bacteria, such as *Salmonella* ( *S. typhimurium* VNP2009) and *Listeria* (CRS‐207), possess inherent capabilities to target tumors and can activate antitumor immunity to inhibit tumor progression [15, 16]. In summary, the development and utilization of oncolytic probiotics for cancer prevention and treatment represent a new direction in cancer biotherapy.

To develop novel intestinal probiotics with therapeutic functions for breast cancer, this study selected *Clostridium butyricum* (CB) and *Akkermansia muciniphila* [17] as candidate strains and analyzed the effects of CB, AKK, and their combination (CB‐AKK) on 4T1 breast tumors in mice. CB is a probiotic that naturally exists in the intestines of humans and animals, capable of producing a substantial amount of short‐chain fatty acids, vitamins, and other metabolites [18]. It has been extensively studied for its roles in regulating gut microbiota, treating diarrhea, inflammatory bowel disease (IBD), and tumors of the urinary and digestive systems, showing significant potential in reshaping the tumor microenvironment, enhancing the efficacy of chemotherapy and immunotherapy, as well as in early diagnosis and treatment efficacy prediction [19, 20, 21]. AKK is a probiotic that naturally degrades mucins and is present in the intestinal walls of healthy individuals [22, 23]. Both live and dead forms of AKK exhibit important physiological regulatory functions and demonstrate significant efficacy in improving inflammation, metabolic diseases (such as obesity, type II diabetes, and cardiovascular diseases), and neurological disorders [24, 25, 26]. Due to the remarkable probiotic functions, stable intestinal colonization, and favorable biosafety of CB and AKK, they have been developed as important pharmaceutical agents and functional foods [27, 28, 29]. Additionally, a well‐established gene editing system for CB and AKK has been developed, laying the groundwork for subsequent genetic modifications [30, 31]. Therefore, CB and AKK may represent the best candidate strains for the development of biotherapy for breast cancer.

To evaluate the functions of CB and AKK in the treatment of breast cancer, this study established a 4T1 breast cancer‐bearing mouse model and analyzed the characteristics of CB, AKK, and their combination (CB‐AKK) in inhibiting breast cancer. The results revealed that oral administration of CB and AKK could reshape the 4T1 breast tumor microenvironment, activate antitumor immunity, and modulate the microbial composition within the intestines of the mice. Furthermore, the combined effect of CB‐AKK was found to be significantly superior to that of single‐strain treatment. This study is the first to demonstrate that the combination of CB and AKK has therapeutic potential against breast cancer, providing novel and promising candidate strains for the advancement of cancer biotherapy for breast cancer treatment.

## 2 Materials and Methods

### 2.1 Bacterial Strains and Culture Conditions

*Akkermansia muciniphila ATCC BAA‐835* [17]: First, the AKK strain stored at −80°C was inoculated onto solid thioglycollate medium (Cat#HB5190‐5, hopebio; Qingdao Hope Bio‐Technology Co. Ltd., Qingdao, China) under anaerobic conditions at 37°C. Single colonies were then picked and inoculated into 5 mL of thioglycollate liquid medium and incubated at 37°C in an anaerobic incubator for 5 days. Subsequently, the culture was transferred to 50 mL thioglycollate medium and continued to be cultured until it reached the logarithmic phase. Finally, the bacteria were collected by centrifugation at 4°C and 5000 rpm. After washing with PBS containing 2.5% glycerol, the bacteria were resuspended to adjust the density to OD600 = 0.8 for further experiments.

*Clostridium butyricum CGMCC0313‐1* (CB): First, the CB strain stored at −80°C was inoculated onto RCM solid medium (Cat#HB0316, hopebio; Qingdao Hope Bio‐Technology Co. Ltd., Qingdao, China) and incubated at 37°C under anaerobic conditions for 2 days. Single colonies were then picked and inoculated into 5 mL of RCM liquid medium and incubated overnight at 37°C in an anaerobic incubator. Subsequently, the culture was transferred to 50 mL of RCM medium and further cultivated for 8 h until it reached the logarithmic phase. Finally, the bacteria were collected by centrifugation at 4°C and 5000 rpm. After washing with PBS containing 2.5% glycerol, the bacteria were resuspended to adjust the density to OD600 = 0.6 for further experiments.

### 2.2 Cell Culture

The 4T1 breast cancer cells stored at −80°C were thawed at 37°C and seeded into T25 culture flasks with 5 mL of RPMI 1640 medium (Cat#c11875500; Gibco, Thermo Fisher Scientific, Chengdu, China) containing 10% fetal bovine serum (FBS) (Cat#11011; Sijiqing, Solarbio Science & Technology Co. Ltd., Beijing, China) and 1% penicillin–streptomycin (Cat#P1400; Solarbio, Solarbio Science & Technology Co. Ltd., Beijing, China). The cultures were placed in a 5% CO_2_ incubator at 37°C and incubated for 2 days. Cells in the logarithmic growth phase were then collected for subsequent experiments.

### 2.3 Animal and Tumor Models

Healthy female BALB/c mice, aged 6–8 weeks and weighing 18 ± 2 g, were purchased from Guizhou Hui‐Tech Co. Ltd. (SCXK, Zhejiang, 2019‐0004). This study was approved by the Experimental Animal Ethics Committee of Guizhou Medical University, with approval number 2200330. All experimental mice were housed at Guizhou Medical University Animal Facilities under specific pathogen‐free (SPF) environmental conditions. The ambient temperature was maintained at 23°C ± 5°C, with a 12‐h light/dark cycle, and the mice had free access to food and water.

On day zero, the mice were injected subcutaneously with 2 × 10^6^ 4T1 breast cancer cells in the axillary region of the forelimb. When the tumor volume reached 100–200 mm^3^. To minimize bias, all animals were randomly assigned to experimental groups by an independent researcher using complete randomization. All the mice were randomly assigned into four groups, with 8 mice per group. The bacterial suspension was administered every 2 days according to the following scheme: 4T1 group, gavage with 200 μL PBS per mouse; AKK group, gavage with 200 μL AKK (1.5 × 10^9^ CFU/mL) per mouse; CB group, gavage with 200 μL CB (1.5 × 10^8^ CFU/mL) per mouse; CB‐AKK group: gavage with 200 μL CB and AKK mixture (AKK 1.5 × 10^9^ CFU/mL, CB 1.5 × 10^8^ CFU/mL). Treatment administration was performed by separate personnel, whereas measurement technicians remained blinded to group allocation throughout the study. The body weight, tumor volume, and survival status of each mouse were recorded every 2 days. Tumor volume was calculated using the formula: tumor volume = 0.5 × *L* × *W* ^2^, where *L* is the longest diameter and *W* is the shortest diameter of the tumor [32]. On Day 20, mice were sacrificed, and the serum, spleens, tumor tissues, and intestinal contents were collected for subsequent analysis.

### 2.4 Intratumoral Bacterial Detection

Fluorescence imaging:To evaluate the ability of CB‐AKK to target 4T1 breast tumors, we labeled the bacteria with two different fluorescent dyes, FITC and ICG, respectively [33, 34]. When the tumor volume reached approximately 300–500 mm^3^, the labeled bacteria were administered via oral gavage to the 4T1 tumor‐bearing mice (bacterial concentrations: CB: 1.5 × 10^8^ CFU; AKK: 1.5 × 10^9^ CFU). After 72 h, the tumors were isolated, and the localization of bacteria within the tumor was assessed using an in vivo fluorescent imaging system (NEWTON 7.0, Vilber).

PCR detection:Approximately 100 mg of fresh tumor tissue was collected, and total DNA was extracted using a genomic DNA extraction kit (Cat#18700es50; YESEN, Yesen BioTechnologies Co. Ltd., Shanghai, China). Primers AKK‐16S‐F/AKK‐16S‐R and AKK‐Amuc_1100‐F/AKK‐Amuc_1100‐R were used to amplify the *16S rRNA* gene and the *Amuc_1100* gene of AKK, respectively. Primers CB‐Pta‐F/CB‐Pta‐R and CB‐14300‐R/CB‐14300‐R were used to amplify the *pta* gene and *Autolytic lysozyme_14,300* gene of CB, respectively.

### 2.5 Spleens Tissue Harvest and Cell Purification

Spleens were collected using autoclaved tools under sterile conditions. The harvested spleens were homogenized and subjected to erythrocyte lysis using the Mouse Red Blood Cell Lysing Kit (Cat#WL2000; BD Pharmingen, Universal Biotech Co. Ltd., Shanghai, China). After lysis of red blood cells, the remaining cells were resuspended in flow cytometry staining buffer (Cat#554656; BD Pharmingen, Universal Biotech Co. Ltd., Shanghai, China) to the final concentration of 1 × 10^6^ cells/mL. Finally, the prepared cells were utilized for flow cytometric analysis.

### 2.6 Flow Cytometry Assay (FCM)

The fresh spleen was crushed to obtain splenocytes, and a red blood cell lysis buffer was applied to eliminate red blood cells. The spleen cells were then stained with fluorescently labeled antibodies, and the samples were incubated at 4°C for 30 min. After incubation, the samples were washed twice with PBS before proceeding to detection on the flow cytometer. The antibodies utilized in the flow cytometry analysis were as follows: APC‐Cy7 Rat Anti‐Mouse CD45(clone 30‐F11, Cat#557659; BD Pharmingen, Universal Biotech Co. Ltd., Shanghai, China, 1:100 dilution) for leukocyte identification, Ms. CD3e APC (clone145‐2C11, Cat#553066; BD Pharmingen, Universal Biotech Co. Ltd., Shanghai, China, 1:100 dilution)for monocyte identification, FITC Rat Anti‐Mouse CD4 (clone RM4‐5; Cat#562091, BD Pharmingen, Universal Biotech Co. Ltd., Shanghai, China, 1:100 dilution) and Ms. CD8a PE (clone 53‐6.7, Cat#553032; BD Pharmingen, Universal Biotech Co. Ltd., Shanghai, China, 1:100 dilution) for the identification of CD4^+^ T and CD8^+^ T cells, respectively. Samples were analyzed using a BD‐FACSCelesta flow cytometer (389150; BD Biosciences) and processed with FlowJo_V10.8.1.

### 2.7 RNA Extraction and qPCR Detection of Tumor Tissue

Approximately 100 mg of tumor tissue samples were obtained and added to 1 mL of Trizol reagent. The samples were then homogenized using a high‐speed, low‐temperature tissue grinder (Cat#SWE‐FP; Servicebio, Servicebio Technology Co. Ltd., Wuhan, China). Total RNA was extracted using an RNA extraction kit (Cat#M5101; New Sai Mei, NCM Biotech, Suzhou, China), and cDNA was synthesized using a reverse transcription kit (Cat#R302‐01; Vazyme, Vazyme Biotech Co. Ltd., Nanjing, China) according to the manufacturer's instructions. The mRNA expression levels of Bax, Bcl‐2, Ki‐67, Caspase‐3, TNF‐α, IL‐6, and IL‐10 cytokines in the tumors were quantified by qPCR, using the β‐actin gene as control. The ΔCT method was employed to calculate the relative expression levels of each gene. The primers used in this study were listed in Table 1.

**TABLE 1:** Primers used in this study.

Prime name	Sequence (5′–3′)	Notes
Bax‐F	CATCCAGGATCGAGCAGGGA	Detect the Bax gene
Bax‐R	CTGCAGCTCCATATTGCTGTCC	
Bcl‐2‐F	GATGGGGTGAACTGGGGGAG	Detect the Bcl‐2 gene
Bcl‐2‐R	GTTATCCTGGATCCAGGTGTGCAG	
Caspase‐3‐F	GATCATAGCAAAAGGAGCAGCTTTGT	Detect the Caspase‐3 gene
Caspase‐3‐R	CTGAATGATGAAGAGTTTCGGCTTTCC	
TNF‐a‐F	CTTCTCATTCCTGCTTGTGGCAG	Detect the TNF‐a gene
TNF‐a‐R	CGAATTTTGAGAAGATGATCTGAGTGTGAG	
Ki‐67‐F	GTATCCAGCTGCCTGTAGTGTCA	Detect the Ki‐67 gene
Ki‐67‐R	CTTCCATCCTCATGATTTCCATCT	
IL‐6‐F	GAGGATACCACTCCCAACAGACC	Detect the IL‐6 gene
IL‐6‐R	GCAAGTGCATCATCGTTGTTCATAC	
IL‐10‐F	ACAACATACTGCTAACCG	Detect the IL‐10 gene
IL‐10‐R	CTGAGGGTCTTCAGCTT	
Amuc_1100‐F	GGCCGCTTAATCTTCAGACGGTTCCTG	Detect the Amuc_1100 gene of Akk
Amuc_1100‐R	AATTAGGACATCGTGGCGGCGGCGGC	
AKK‐16S‐F	AGAGGTCTCAAGCGTTGTTC	Detect the 16S gene of Akk
AKK‐16S‐R	CACCGTTTACTGCCAGGACTA	
515F	GTGCCAGCMGCCGCGGTAA	Detect bacterial housekeeping genes
806R	GGACTACHVGGGTGTCTAAT	
CB‐14360‐F	GGATCCATGTTGCTTTGGTTTAGTTTATTCAAT	Detect the Autolytic lysozyme gene of CB
CB‐14360‐R	TTATTGATTTATTAGGCAGCCTTCATT	
CB‐Pta‐F	CCTTTAGGGGTAGAAGCAAAAAGT	Detect the Pta gene of CB
CB‐Pta‐R	GTCATTCTTTCTAAGATAAACTC	
B‐Actin‐F	GTGGTACCACCAGACAGCAC	Reference gene
B‐Actin‐R	ATCACTATTGGCAACGAGCGG	

### 2.8 Histological Evaluation and Immunohistochemistry

Hematoxylin and eosin (H&E) sections and immunohistochemical staining of tumor tissues were performed by Wuhan Huayan Biotechnology Co. Ltd. Tumor cross sections were fixed with paraformaldehyde, followed by paraffin embedding, and stained with hematoxylin–eosin. The tumor tissue sections were incubated with anti‐CD4 (Cat#11‐0040‐82; Invitrogen), anti‐CD8 (Cat#bs‐0648R; Bioss), anti‐Bax (Cat#50599‐2‐IG; Proteintech) and anti‐Ki‐67 (Cat#28074‐1‐AP; Proteintech) antibodies for immunohistochemistry analysis. The antibodies were supplied by Huayan Technology Company. To minimize bias, all tumor tissue samples were randomly coded by an independent researcher prior to processing, ensuring that the technicians performing H&E staining and IHC were blinded to the experimental groups. The IHC staining results were analyzed using ImageJ (US National Institutes of Health, Bethesda, MD). The histopathological evaluation was conducted in a blinded manner following the published standard [35].

### 2.9 Enzyme‐Linked Immunosorbent Assay (ELISA)

Serum cytokines: Whole blood was collected from the mice's eyeballs, and serum was separated by centrifugation at 3000 rpm. The levels of TNF‐α (Cat#EK282; MULTI SCIENCES, MultiSciences Biotech Co. Ltd., Hangzhou, China), IL‐6 (Cat#EK206; MULTI SCIENCES, MultiSciences Biotech Co. Ltd., Hangzhou, China) and IL‐10 (Cat#EK210; MULTI SCIENCES, MultiSciences Biotech Co. Ltd., Hangzhou, China) in the serum were measured using an ELISA kit according to the manufacturer's instructions.

Tumor tissue cytokines: Equal weights of tumor tissue were obtained and homogenized using a high‐speed, low‐temperature tissue grinder (Cat#SWE‐FP; Servicebio, Servicebio Technology Co. Ltd., Wuhan, China). Total protein quantification of the tumor homogenate was performed using a BCA protein assay kit (Cat#PCoo20‐500; Solarbio, Solarbio Science & Technology Co. Ltd., Beijing, China), and the levels of TNF‐α, IL‐6, and IL‐10 in the tumor homogenate were measured using an ELISA kit (ibid) following the manufacturer's instructions.

### 2.10 16S rRNA Sequencing

To assess the bacterial community structure in intestinal feces, bacterial 16S rRNA sequencing was performed in this study (Majorbio). Total DNA was extracted from mouse fecal samples using the FastPure Stool DNA Isolation Kit (MJYH, shanghai, China). The bacterial 16S rRNA V3‐V4 region was amplified by PCR using the primers 338F (5′‐ACTCCTACGGGAGGCAGCAG‐3′) and 806R (5’‐GGACTACHVGGGTWTCTAAT‐3′). Bioinformatic analysis of the gut microbiota was carried out using the Majorbio Cloud platform (https://cloud.majorbio.com). Based on the ASV data, rarefaction curves and alpha diversity metrics, including observed ASVs, Chao1 richness, Shannon index, and Good's coverage, were computed using Mothur version 1.30.2.

### 2.11 Cell Analysis

To analyze the effects of CB and AKK metabolites on cell viability and apoptosis, the supernatants from the fermentation cultures of CB and AKK during the logarithmic growth phase were collected. After being filtered through a bacterial filter, the supernatants were incubated with cultured 4T1 cells (2 × 10^4^) in the following groups: CT group: 30 μL of sodium thioglycolate medium + 70 μL of cell culture medium; CB group: 30 μL of CB supernatant + 70 μL of cell culture medium; AKK group: 30 μL of AKK supernatant + 70 μL of cell culture medium; CB‐AKK group: 30 μL of CB supernatant + 30 μL of AKK supernatant + 40 μL of cell culture medium. After the cultures were incubated with the 4T1 cells for 24 h, cell viability and apoptosis were assessed using a microplate reader and flow cytometry, respectively. For the analysis of cell viability, 10 μL of MTT solution was added, and the cultures were further incubated for an additional 4 h, after which 110 μL of Formazan solution was added to dissolve the formed formazan crystals. Finally, the absorbance at 490 nm was measured using a microplate reader (Cat# 168‐1130; Bio‐Rad iMark). Cell viability was calculated as follows: Cell viability (%) = (Experimental group A490/Control group A490) × 100%. For the analysis of cell apoptosis, the same method as described in Section 2.6 was utilized. The experiments were repeated at least three times.

### 2.12 Western Blotting

Bacterial culture, cell culture, and experimental grouping were conducted as described above. When the 4T1 cells reached a concentration of 2.6 × 10^6^, 5 mL of the supernatant was added and incubated for 48 h in a cell culture incubator maintained at 5% CO_2_ at 37°C. Subsequently, the cells were collected by centrifugation and washed with prechilled PBS at 4°C. The cells were then lysed in RIPA lysis buffer containing PMSF and protease inhibitors (Nanjing Kaiji Biological Development Co. Ltd., Nanjing, China). The total protein concentration was quantified using a BCA protein assay kit (Cat#PCoo20‐500; Solarbio, Solarbio Science & Technology Co. Ltd., Beijing, China). Following this, the samples were incubated with the primary antibodies for Caspase‐3 (clone JE75‐05, Cat#ET1602‐39; HuaBio, Huayuan Biotechnology Co. Ltd., Hangzhou, China, 1:2000 dilution), Bax (clone SZ3‐07, Cat#ER0907; HuaBio, Huayuan Biotechnology Co. Ltd., Hangzhou, China, 1:2000 dilution), and Bcl‐2 (clone JF104‐8, Cat#ET1702‐53; HuaBio, Huayuan Biotechnology Co. Ltd., Hangzhou, China, 1:2000 dilution) at 4°C overnight, followed by washing with TBST five times. After washing, the samples were incubated with rabbit secondary antibody at room temperature for 1 h, and then washed with TBST another five times before detection using a protein exposure instrument (Cat#12009077; Bio‐Rad GelDoc Go).

### 2.13 Statistical Analysis

One‐way ANOVA and nonparametric tests were applied for the analysis of animal data in this study. All results were expressed as mean ± standard error. *p* values were adjusted for multiple comparisons using the Bonferroni correction. Statistical significance was set at *p* < 0.05 after correction. Statistical analyses of all data were performed using GraphPad Prism 9.4.0. (****p < 0.0001, ***p < 0.001, **p < 0.01, *p < 0.05.).

## 3 Results

### 3.1 Oral Probiotics CB‐AKK Inhibit 4T1 Breast Tumor Progression and Improve Survival Rate in Mice

**FIGURE 1:**
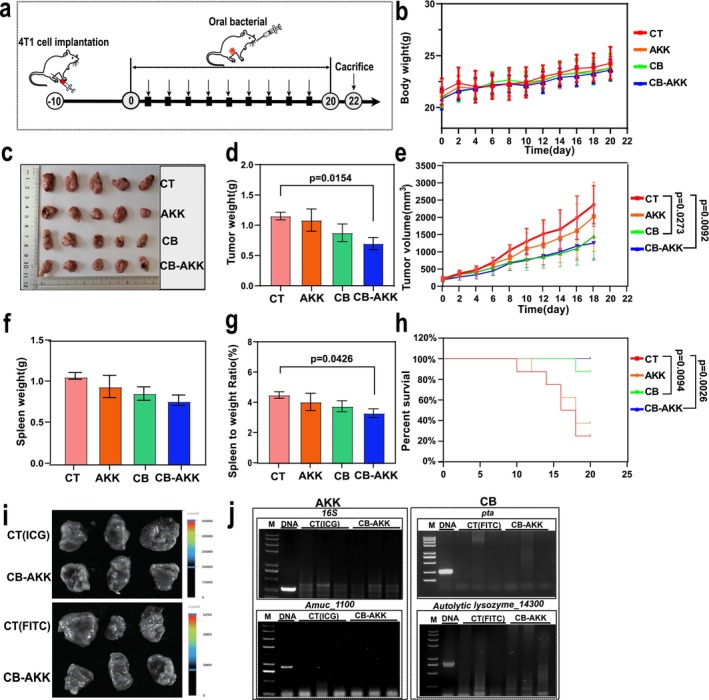
Oral probiotics CB‐AKK inhibit the progression of 4T1 breast tumors and improve survival rate in mice. (a) Schematic illustration of the experimental design. (b) Body weight measurements. (c–e) Tumor images, tumor weights, and tumor volumes. (f–h) Spleen weights, spleen‐to‐body weight ratios, and survival rates. (i) Fluorescent images of mouse tumors after 72 h of oral administration. (j) PCR detection of AKK‐specific genes and CB‐specific genes. AKK: 16S rRNA (260 bp), Amuc_1100 (902 bp); CB: Pta genes (386 bp), Autolytic lysozyme_14,300 gene (797 bp); The templates are: DNA (genomic DNA of AKK or CB); CT (genomic DNA of tumors in control group); CB‐AKK (genomic DNA of tumors in CB‐AKK treatment group). *n* = 8. Data are presented as mean ± SEM.

To evaluate the therapeutic effects of probiotics CB and AKK on distal intestinal tumors in breast cancer, gastric gavage of CB, AKK, and the combination of CB‐AKK was performed in 4T1 breast cancer‐bearing mice, and their impact on tumor progression and survival rates was analyzed (Figure 1a). No significant changes in body weight were observed among the groups following probiotic treatment (Figure 1b). However, notable reductions in tumor volume, tumor weight, and spleen‐to‐body weight ratio were recorded. Specifically, tumor weights were reduced by 6%, 24%, and 40% in the AKK, CB, and CB‐AKK groups, respectively, whereas tumor volumes decreased by 15%, 40%, and 47%, and the spleen‐to‐body weight ratios decreased by 10%, 16%, and 26%. Additionally, significant changes in survival rates among the groups of mice were noted at the endpoint of the treatment. The survival rate in the CB‐AKK group was 100%, significantly higher than that of the control group and the single‐bacterium treatment groups. These results indicated that oral probiotics CB and AKK possess the ability to inhibit the progression of 4T1 breast tumors; however, the combined effect of CB‐AKK is far superior to that of the single‐strain treatments.

Studies have shown that certain oncolytic bacteria, such as EcN and Salmonella, can actively target tumors to suppress or influence tumor progression [36]. To evaluate the ability of CB‐AKK to actively target tumors, we labeled CB‐AKK with two fluorescent dyes, FITC and ICG. After oral gavage for 72 h, tumor tissues were isolated from the mice, and a fluorescence imaging system was utilized to observe bacterial targeting within the tumors. However, the detection results indicated that no fluorescence was observed in the tumor tissues (Figure 1i). Subsequently, genomic DNA was extracted from the tumors, and CB‐specific primers and AKK‐specific primers were employed to amplify the specific genes of CB (*pta* gene and *Autolytic lysozyme_14,300* gene) and AKK (*16S rRNA* gene and *Amuc_1100* gene), respectively. Nevertheless, the presence of CB and AKK within the tumors was also not detected (Figure 1j). In conclusion, these results indicated that CB and AKK do not actively target breast tumors and cannot treat breast cancer through intratumoral colonization; their anticancer effects may be mediated through mechanisms such as the modulation of the body's immune responses.

### 3.2 Oral Probiotics CB‐AKK Regulate Intestinal Microbial Composition and Abundance

**FIGURE 2:**
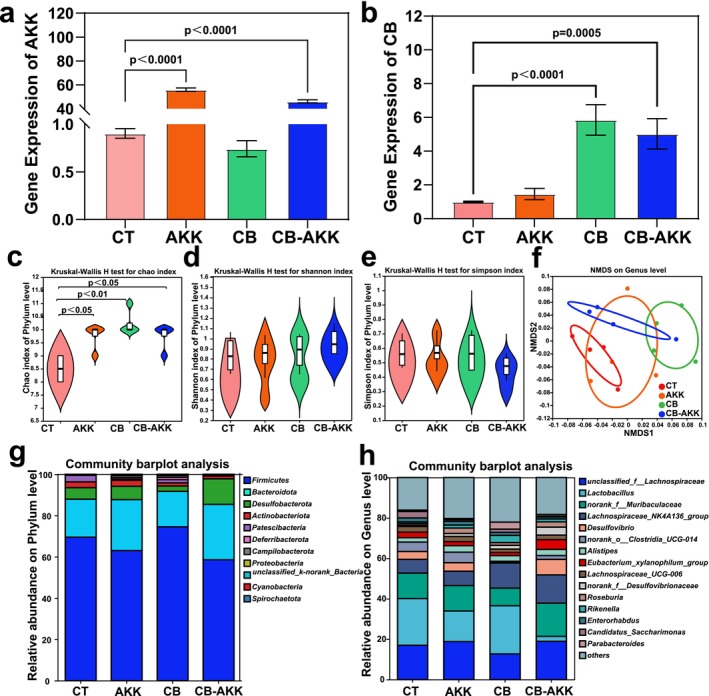
Oral probiotics CB‐AKK regulate intestinal microbial composition and abundance in 4T1 breast cancer‐bearing mice. (a) qPCR detection of AKK content in the gut. (b) qPCR detection of CB content in the gut. (c–e) α‐diversity indices based on 16S rRNA gene sequencing, including Chao 1 index, Shannon index, and Simpson index. (f) NMDS analysis of β‐diversity based on 16S rRNA gene sequencing. (g, h) Stacked bar charts showing the percentages of the top 11 bacterial phyla at the phylum level and the top 15 bacterial genera at the genus level. Data are presented as mean ± SEM.

Intestinal flora plays a crucial role in the occurrence, development, and treatment of tumors [37, 38]. Under normal circumstances, the gastrointestinal barrier and the digestion of gastric and intestinal fluids significantly hinder the ability of live bacteria to reach the intestine [17]. To determine the capacity of oral CB‐AKK to reach the intestine, qPCR was first utilized to detect the relative contents of CB and AKK in mouse feces. As shown in (Figure 2a,b), oral administration of CB‐AKK significantly increased the contents of CB and AKK in feces, with their relative abundances increasing by 5‐fold and 51‐fold, respectively.

Subsequently, 16S rRNA sequencing was employed to analyze the effects of oral probiotics CB‐AKK on intestinal microbiota in mice. The results indicated that oral administration of CB‐AKK significantly altered the composition and abundance of intestinal microbes in mice (Figure 2c–f). Phylum‐level species abundance analysis revealed that the abundance of *Firmicutes* was significantly reduced in the CB‐AKK group (Figure 2g), whereas the abundance of *Bacteroidota* and *Desulfobacterota* was significantly increased. Genus‐level abundance analysis showed that *Lachnospiraceae* and *Alistipes* increased in the CB‐AKK group; these two genera have been shown to play a beneficial role in cancer immunotherapy by regulating the tumor microenvironment [39, 40]. However, the abundances of both *Lactobacillus* and *Clostridia* decreased significantly in the CB‐AKK group. We hypothesized that the introduction of CB and AKK may have disrupted the homeostasis of the gut microbiota, leading to a decline in the relative abundances of certain microbiota. This change may be attributed to competition between CB, AKK, and other bacteria, or due to the effects of metabolites produced by CB and AKK on the growth environment of other bacteria. In conclusion, omics analysis suggests that oral administration of CB‐AKK can modulate the intestinal microbial composition and abundance.

### 3.3 Oral Probiotics CB‐AKK Activate Anti‐Tumor Immunity, Increase the Production of TNF‐α

**FIGURE 3:**
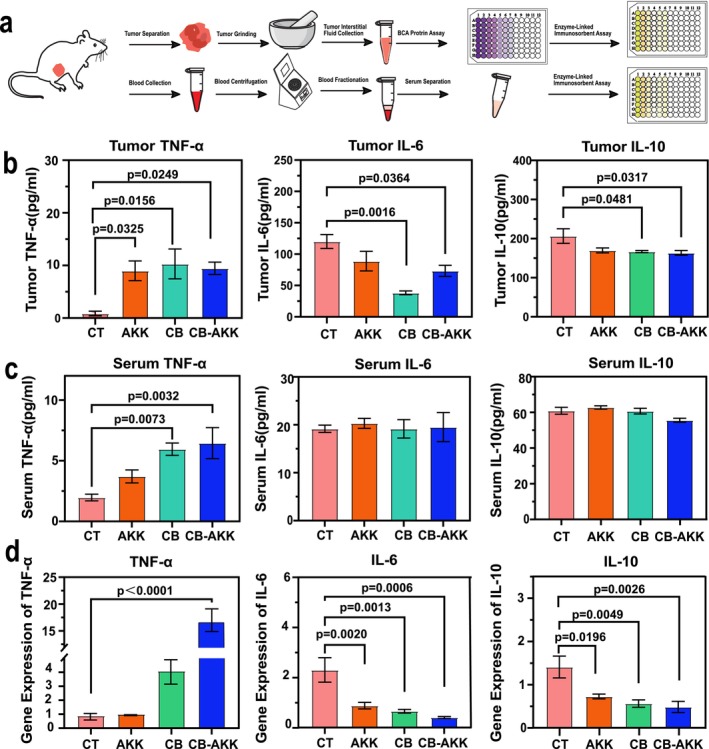
Oral probiotics CB‐AKK activate antitumor immunity, increase the production of TNF‐α in mice. (a) Schematic illustration of the ELISA experimental design for tumor and serum samples. (b) ELISA detection of TNF‐α, IL‐6, and IL‐10 concentrations in tumor samples. (c) ELISA detection of TNF‐α, IL‐6, and IL‐10 concentrations in serum samples. (d) qPCR detection of TNF‐α, IL‐6, and IL‐10 gene expression in tumors. Data are presented as mean ± SEM.

To evaluate the effects of oral probiotics CB and AKK on the immune response in 4 T1 tumor‐bearing mice, we measured the changes in levels of tumor necrosis factor (TNF‐α), IL‐6, and IL‐10 in both the tumors and serum of the mice. Analysis of tumor tissues indicated that, compared to the control group, the concentrations of TNF‐α were significantly elevated in the CB group, AKK group, and CB‐AKK combined group, whereas the concentrations of IL‐6 and IL‐10 were significantly reduced (Figure 3b).

Serum analysis revealed that, compared to the control group, although there were no significant changes in the concentrations of IL‐6 and IL‐10, TNF‐α levels were significantly increased in both the CB group and the CB‐AKK combined group (Figure 3c). Furthermore, we also analyzed the expression of TNF‐α, IL‐6, and IL‐10 genes in tumor tissues, which were consistent with the ELISA results, with the most significant changes observed in the CB‐AKK combined group improved by 19 times (Figure 3d). In conclusion, the data indicated that oral probiotics CB‐AKK can activate antitumor immunity in breast cancer mice.

### 3.4 Oral Probiotics CB‐AKK Enhance CD8 ^+^ T Cell‐Mediated Anti‐Tumor Immunity

**FIGURE 4:**
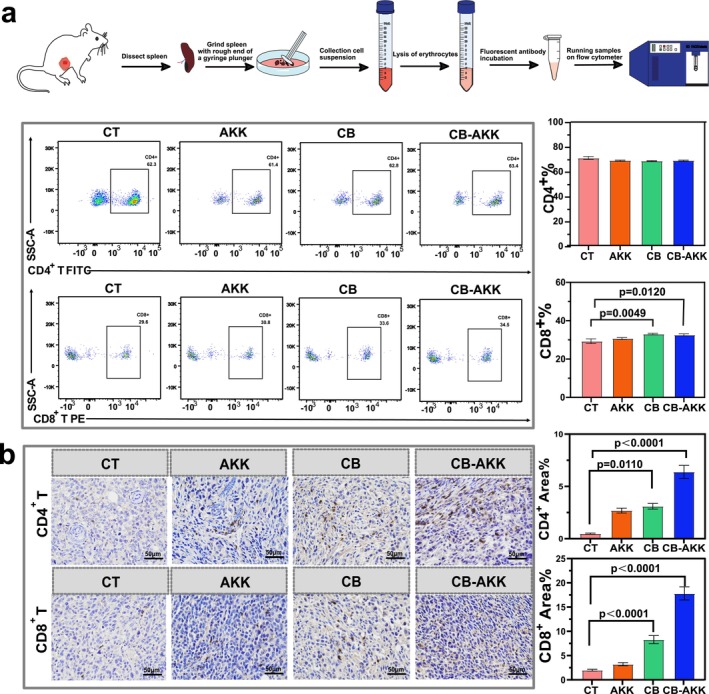
Oral probiotics CB‐AKK enhance CD8^+^ T cell‐mediated antitumor immunity. (a) Flow cytometric analysis of the proportions of CD4^+^ T and CD8^+^ T cells in the spleen. (b) Immunohistochemical analysis of the proportions of CD4^+^ T and CD8^+^ T cells in the tumor. Data are presented as mean ± SEM.

The spleen is recognized as the most important immune organ in the body and serves as a site for the aggregation and maturation of lymphocytes, including T cells [41, 42]. To evaluate the impact of oral CB‐AKK on immune function, the spleens were first isolated from mice, and the proportions of CD4^+^ T and CD8^+^ T cells were analyzed using flow cytometry. The results indicated that no significant differences in the proportions of CD4^+^ T cells were observed among the groups compared to the control; however, the proportion of CD8^+^ T cells was found to be increased in all test groups, with the CB group showing an increase of 4% (*p* = 0.0049) and the CB‐AKK group exhibiting an increase of 2.6% (*p* = 0.0120) (Figure 4a).

In addition, we further assessed the changes in CD4^+^ T and CD8^+^ T cells in the tumor microenvironment using immunohistochemistry. It was observed that both CD4^+^ T and CD8^+^ T cells increased in all treatment groups, with the CB‐AKK combination group showing the most significant increase compared to the control group. Specifically, CD4^+^ T cells were increased 13.3‐fold and CD8^+^ T cells were increased 8.9‐fold (*p* < 0.0001) (Figure 4b). Combining the results from flow cytometry and immunohistochemistry, it was found that the increase in CD8^+^ T cells in the CB‐AKK combination group was particularly pronounced. Given that CD8^+^ T lymphocytes are key effector cells in the antitumor immune response, this significant increase indicates that CB‐AKK treatment substantially enhances the antitumor immune response in mice.

### 3.5 Oral Probiotics CB‐AKK Activate the Bcl‐2/Bax Pathway and Promote Tumor Apoptosis

**FIGURE 5:**
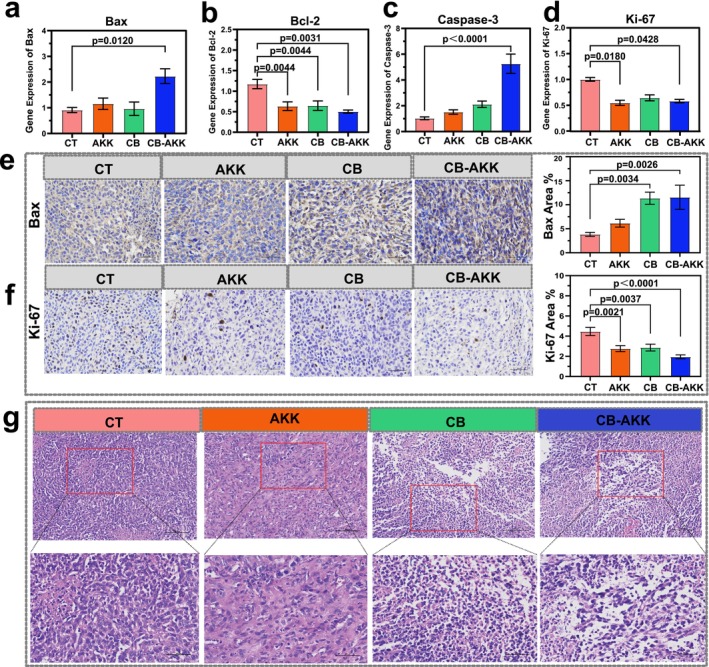
Oral probiotics CB‐AKK activate the Bcl‐2/Bax pathway and promote tumor apoptosis. (a–d) qPCR detec‐tion of gene expression for Bax, Bcl‐2, Caspase‐3, and Ki‐67 in tumor tissue. (e, f) Immunohistochemical detection of Bax and Ki‐67 protein. (g) HE staining of tumor tissue at 200× and 400× magnification. Data are presented as mean ± SEM.

The Bcl‐2/Bax signaling pathway is recognized as one of the key pathways influencing the apoptosis of breast cancer cells [43, 44]. In this pathway, Bcl‐2 is a critical oncogene that significantly inhibits apoptosis, whereas Bax antagonizes Bcl‐2 and promotes apoptosis [45]. To elucidate the mechanism by which oral CB‐AKK probiotics inhibit the progression of 4T1 breast tumors, qPCR and immunohistochemistry were performed to assess the expression of genes associated with the Bcl‐2/Bax pathway within tumor tissues. Our findings indicated that, following CB‐AKK combination treatment, the expression of the Bax gene was significantly increased (*p* = 0.0120), whereas the expression of the Bcl‐2 gene was significantly decreased (*p* = 0.0031) (Figure 5a,b).

Caspase‐3 is recognized as the most critical apoptosis executor in the caspase pathway, and its function can be inhibited by Bcl‐2, which plays a pivotal role in apoptosis [46]. Additionally, Ki‐67 is associated with the proliferative capacity of tumor cells; therefore, changes in both Caspase‐3 and Ki‐67 gene expression were also analyzed. It was found that Caspase‐3 was upregulated 4.5‐fold in the CB‐AKK group compared with the control group (*p* < 0.0001), and Ki‐67 decreased by 0.5‐fold (*p* = 0.0428), indicating that CB‐AKK treatment inhibited tumor cell proliferation and significantly promoted tumor cell apoptosis. Furthermore, histopathological changes in tumors across each group after probiotic treatment were also analyzed, which were consistent with the aforementioned gene detection results. In conclusion, based on the results of qPCR, immunohistochemistry, and histopathological analysis, it was found that oral probiotics CB‐AKK could activate the Bcl‐2/Bax pathway to effectively inhibit the proliferation of 4T1 tumor cells and promote the apoptosis of tumor cells.

### 3.6 Cell Experiments Indicated That CB‐AKK Metabolites Regulate the Blc‐2/Bax Pathway to Induce Apoptosis of 4T1 Tumor Cells

**FIGURE 6:**
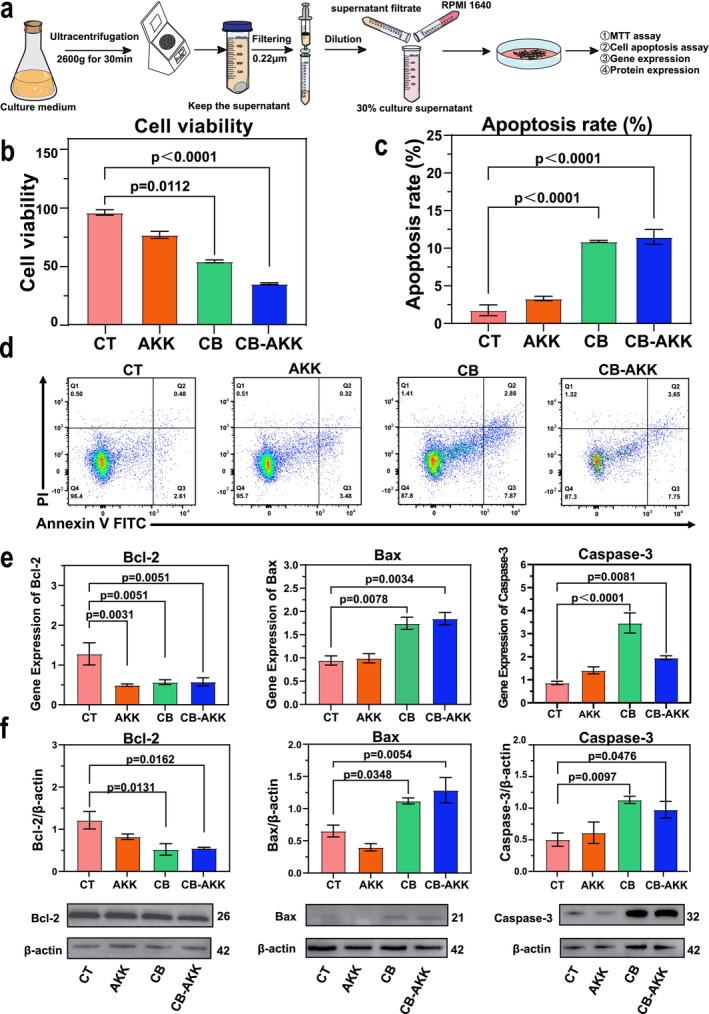
The effects of CB‐AKK metabolites on 4T1 tumor cells. (a) Schematic il‐lustration of the preparation and functional analysis of CB‐AKK metabolites. Bacte‐ria were cultured until the logarithmic phase, then centrifuged at 4600 *g* for 30 min. The supernatant was collected and filtered. The filtered supernatant was diluted to 30% of its original concentration using RPMI 1640 cell culture medium and applied to 4T1 cells. Cell viability, apoptosis, and the expression of proteins and genes were subsequently measured. (b) 4T1 cell viability assay using MTT. (c, d) Apoptosis assay for 4T1 cell conducted via FCM. (e) Gene expression of Bcl‐2, Bax, and Caspase‐3 in 4T1 cells treated with probiotic metabolites of CB, AKK and CB‐AKK. (f) Western blotting de‐tection of Bcl‐2, Bax, and Caspase‐3 protein. Data are presented as mean ± SEM.

To further elucidate the mechanism by which CB‐AKK inhibits the progression of 4T1 breast tumors, the metabolites of CB, AKK, and CB‐AKK were incubated with 4T1 tumor cells in vitro, and the effects of probiotic metabolites on the viability and apoptosis of 4T1 cells were analyzed using MTT assay and flow cytometry. It was found that, compared to the control group, probiotic metabolites significantly reduced cell viability and enhanced cell apoptosis, with the most pronounced effect observed in the CB‐AKK combined treatment group, which resulted in a cell viability decrease of 63% and an apoptosis rate increase of 6.6‐fold (Figure 6b,c).

Subsequently, qPCR and Western blotting (WB) were utilized to analyze the expression of Bcl‐2/Bax apoptosis‐related genes at both mRNA and protein levels. It was observed that the expression of the Bcl‐2 gene decreased by 45% at the mRNA level and 43% at the protein level in the CB‐AKK group, whereas the expression of the Bax gene increased by 1.7‐fold and 1.9‐fold, respectively. Additionally, the expression of the Caspase‐3 gene was also analyzed, revealing that the expression of this gene increased by 2.3‐fold at the mRNA level and 1.9‐fold at the protein level (Figure 6e,f). In conclusion, the results of our cell experiments indicated that the metabolites of probiotics CB‐AKK could induce apoptosis of 4T1 breast tumor cells by activating the Bcl‐2/Bax pathway.

## 4 Discussion

The primary purpose of this study is to evaluate the functions of intestinal probiotics CB and AKK in the treatment of distal intestinal tumors, specifically breast cancer, and lay a foundation for the development of new biotherapy techniques for breast cancer. This study found that oral administration of the CB‐AKK combined strain significantly alleviated tumor progression in 4T1 breast cancer mice, and the combined effect of the two probiotics was far superior to the individual treatments with CB or AKK, indicating that the probiotic functions of CB and AKK had a significant synergistic effect.

Furthermore, this study demonstrated that oral administration of the CB‐AKK combined bacteria significantly increased the levels of TNF‐α in both tumor tissues and serum, whereas simultaneously reducing the levels of IL‐6 and IL‐10. Additionally, the levels of T lymphocytes in the spleen were significantly elevated, particularly with a notable increase in CD8^+^ T lymphocytes. This finding suggests that CB‐AKK possesses favorable antigenic properties and can activate the body's immune response. Given that CD8^+^ T lymphocytes are critical effector cells in the body's antitumor immune response, capable of directly killing tumor cells and modulating the functions of other immune cells through cytokine secretion, the significant increase in the proportion of CD8^+^ T cells reflects an enhancement in local immune activity within the tumor tissue [47, 48, 49]. This implies that CB‐AKK treatment significantly activates the antitumor immune system in mice.

**FIGURE 7:**
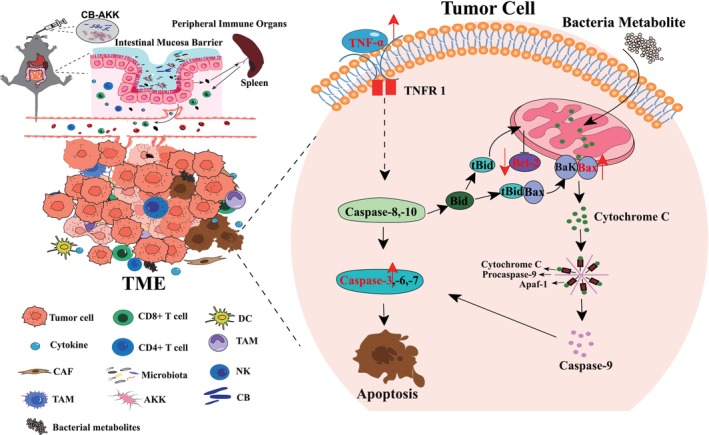
Mechanistic illustration of the effects of CB‐AKK on 4T1 breast cancer.

The Bcl‐2/Bax signaling pathway and Caspase‐dependent apoptosis pathway play an important role in promoting the occurrence and development of tumor cells [43, 50, 51, 52]. Bcl‐2 and Bax are a pair of antagonistic genes, and the ratio between Bcl‐2 and Bax is a key factor in the regulation of apoptosis [53, 54]. Typically, the expression levels of Bcl‐2 and Bax are relatively stable; when Bax is overexpressed, the Bax/Bax homodimer increases, initiating apoptosis [54]. Conversely, when Bcl‐2 is overexpressed, the Bax/Bax homodimer dissociates, forming a more stable Bcl‐2/Bax heterodimer that inhibits apoptosis [55]. In this study, both in animal experiments and in vitro cell experiments, we observed a decrease in Bcl‐2 expression and an increase in Bax expression, thereby altering the balance between Bcl‐2 and Bax. The reduction of the Bcl‐2/Bax ratio induces mitochondrial membrane damage of tumor cells, leading to the massive release of Cytochrome C and ultimately enhancing the expression and activity of Caspase‐3 (Figure 7). Moreover, after CB‐AKK treatment, a significant number of activated T lymphocytes (CD4^+^ T and CD8^+^ T) were produced in the spleen and tumor cells of the mice. The activated T lymphocytes secrete a large amount of TNF‐α, which binds to receptors on the surfaces of tumor cells to activate the Caspase signaling pathway, further enhancing the activity and expression of Caspase‐3. Activated Caspase‐3 is regarded as the most critical apoptotic protein, ultimately promoting tumor cell apoptosis (Figure 7).

CB is a probiotic found in the human gut that plays a vital role in maintaining intestinal barrier function, promoting the health of intestinal epithelial cells, and inhibiting intestinal inflammation and tumor formation [56]. Its metabolites, such as short‐chain fatty acids, succinate, indole and its derivatives, and trimethylamine N‐oxide, can enter the bloodstream through the mesentery and reach tumors and immune organs, further activating the body's immune response [57, 58]. CB has been developed into pharmaceuticals, dietary supplements, and feed additives for the treatment of gut‐related diseases in both humans and animals [59, 60]. AKK is also a probiotic present in the human gut, primarily colonizing the intestinal mucosa and relying on mucin as its main energy source [61, 62, 63]. The components of AKK (e.g., Amuc_1100) and its metabolites play significant roles in restoring gut microbiota balance, repairing damaged intestinal mucosa, maintaining the integrity of the intestinal barrier, improving metabolism, and reducing inflammation levels [64, 65]. Our study found that, in addition to the aforementioned functions, the combined CB‐AKK demonstrates significant efficacy in the treatment of distal tumors, specifically breast cancer, providing a basis for the development of its new therapeutic potential.

Although animal experiments and in vitro cell studies indicate that the combined effect of CB‐AKK probiotics is significantly superior to single‐strain treatment, several issues remain that require further in‐depth analysis. For instance, CB and AKK belong to different types of intestinal probiotics, and their individual effects can vary greatly among different individuals. Factors such as the ratio of CB to AKK, the colonization sites of CB and AKK in the gut, colonization ratios, and survival times may all influence therapeutic efficacy against breast cancer. Additionally, although literature reports that short‐chain fatty acids (such as butyrate) produced by CB, as well as components of AKK (e.g., Amuc_1100), play important roles in anti‐inflammatory responses, regulation of gut microbiota composition, and modulation of immune and metabolic functions [66], the complex nature of CB‐AKK's metabolites remains an unresolved issue. It is not yet clear which specific metabolic products predominantly contribute to the inhibition of 4T1 breast tumor progression or whether only the composite metabolic products of CB‐AKK possess functional properties, and this speculation still needs to be validated by extensive experimentation.

In conclusion, probiotics play a significant role in promoting human health, and utilizing probiotics for the prevention and treatment of cancer represents a novel area of research. CB and AKK are naturally occurring probiotics in the human body with favorable biosafety profiles [67]. Exploring the potential of the CB‐AKK combination in cancer prevention and developing it into a new live microbial preparation and functional food may offer new biotherapeutic strategies and approaches for the treatment of various cancers, including breast cancer.

## Author Contributions

**Xiaowei Li:** methodology, writing – original draft, data curation, validation, investigation. **Dengxiong Hua:** methodology, validation. **Daoyan Wu:** methodology, resources. **Wei Hong:** project administration, conceptualization. **Yingqian Kang:** software. **Lei Tang:** formal analysis. **Qin Yang:** software. **Xinxin Wang:** software, investigation. **Boyan Li:** resources. **Renmin Li:** funding acquisition. **Zhenghong Chen:** conceptualization, visualization. **Guzhen Cui:** conceptualization, supervision, project administration, writing – review and editing.

## Consent

The authors have nothing to report.

## Conflicts of Interest

The authors declare no conflicts of interest.

## Data Availability

Data are contained within the article.
